# Complete Genome Sequence of *Spiroplasma corruscae* EC-1^T^ (DSM 19793), a Bacterium Isolated from a Lampyrid Beetle (*Ellychnia corrusca*)

**DOI:** 10.1128/genomeA.00964-17

**Published:** 2017-09-14

**Authors:** Yi-Ming Tsai, Wen-Sui Lo, Chih-Horng Kuo

**Affiliations:** Institute of Plant and Microbial Biology, Academia Sinica, Taipei, Taiwan

## Abstract

*Spiroplasma corruscae* EC-1^T^ (DSM 19793) was isolated from the gut of a lampryid beetle (*Ellychnia corrusca*) collected in Beltsville, MD, USA, in 1983. Here, we report the complete genome sequence of this bacterium to facilitate the investigation of its biology and the comparative genomics among *Spiroplasma* species.

## GENOME ANNOUNCEMENT

*Spiroplasma corruscae* is known to be associated with firefly beetles (Coleoptera: Lampyridae) and tabanid flies (Diptera: Tabanidae) in North America (1). EC-1^T^ was isolated from the gut of a lampryid beetle (*Ellychnia corrusca*) collected in Beltsville, MD, USA, in 1983 and was designated the representative of group XIV within the genus. To facilitate future investigation on the biology of this bacterium, as well as to improve the taxon sampling of available *Spiroplasma* sequences for comparative genomics and evolutionary studies (2), we determined the complete genome sequence of *S. corruscae* EC-1^T^.

The strain was acquired from the German Collection of Microorganisms and Cell Cultures (catalog number DSM 19793). The freeze-dried sample was processed according to the manufacturer’s instruction and cultured in the M1D medium (3) prior to DNA extraction using the Wizard Genomic DNA purification kit (Promega, USA). PCR and Sanger sequencing were performed to verify that the 16S rRNA gene sequence matched the reference record (GenBank accession number AY189128) (4).

The procedures for genome sequencing, assembly, and annotation were based on those described in our previous studies (5–14). Briefly, we utilized the Illumina MiSeq platform to obtain 301-bp sequencing reads from one paired-end library with approximately 550-fold coverage. The initial *de novo* assembly was performed using Velvet version 1.2.10 (15). Subsequently, PAGIT version 1 (16) was used to assist an iterative process for improving the assembly. For each iteration, the raw reads were mapped to the assembly using the Burrows-Wheeler Alignment (BWA) tool version 0.7.12 (17), programmatically checked using the mpileup program in SAMtools package version 1.2 (18), and visually inspected using Integrative Genomics Viewer (IGV) version 2.3.57 (19). Polymorphic sites and gaps were corrected based on the mapped reads. The process was repeated until the complete genome sequence was obtained. The programs RNAmmer (20), tRNAscan-SE (21), and Prodigal (22) were used for gene prediction. The gene names and product descriptions were first annotated based on the homologous genes in other *Spiroplasma* genomes (5–14) as identified by OrthoMCL (23). Subsequent manual curation was based on the information obtain from the BlastKOALA tool (24) and BLASTp (25) searches against the NCBI nonredundant database (26). Putative clustered regularly interspaced short palindromic repeats (CRISPRs) were identified using CRISPRFinder (27).

The complete genome sequence of *Spiroplasma corruscae* EC-1^T^ consists of one circular chromosome (1,175,400 bp; 25.4% G+C) and one plasmid (29,239 bp; 23.9% G+C). The first version of annotation includes one set of 16S-23S-5S rRNA genes, 29 tRNA genes (covering all 20 amino acids), 1,039 protein-coding genes, and one pseudogene. No putative plectroviral sequence or CRISPR element was found.

### Accession number(s).

The complete genome sequence of *Spiroplasma corruscae* EC-1^T^ has been deposited at DDBJ/EMBL/GenBank under the accession numbers CP022535 (chromosome) and CP022536 (plasmid).
